# Ovarian Cancer Biomarkers: A Focus on Genomic and Proteomic Findings

**DOI:** 10.2174/138920207782446142

**Published:** 2007-08

**Authors:** Andrea Tinelli, Daniele Vergara, Roberta Martignago, Giuseppe Leo, Antonio Malvasi, Raffaele Tinelli, Santo Marsigliante, Michele Maffia, Vito Lorusso

**Affiliations:** 1 Department of Obstetrics and Gynecology, “Vito Fazzi” Hospital, Lecce, Italy; 2 National Nanotechnology Laboratory (NNL), CNR-INFM, University of Lecce, Italy; 3 Department of Biological and Environmental Sciences and Technologies, Laboratory of Human Anatomy, University of Lecce, Italy; 4 Molecular Biology and Experimental Oncology Lab, Oncological Hospital, Lecce, Italy; 5 Department of Obstetrics and Gynecology, “Santa Maria” Hospital, Bari, Italy; 6 Department of Science, Biotechnology and Biology Environment (DiSTeBa), University of Lecce, Lecce, Italy; 7 Department of Oncology, “Vito Fazzi” Hospital, Lecce, Italy

**Keywords:** Ovarian cancer, biomarkers, proteomics, genomics, management.

## Abstract

Among the gynaecological malignancies, ovarian cancer is one of the neoplastic forms with the poorest prognosis and with the bad overall and disease-free survival rates than other gynaecological cancers; several studies, analyzing clinical data and pathological features on ovarian cancers, have focused on the identification of both diagnostic and prognostic markers for applications in clinical practice. High-throughput technologies have accelerated the process of biomarker discovery, but their validity should be still demonstrated by extensive researches on sensibility and sensitivity of ovarian cancer novel biomarkers, determining whether gene profiling and proteomics could help differentiate between patients with metastatic ovarian cancer and primary ovarian carcinomas, and their potential impact on management.

Therefore, considerable interest lies in identifying molecular prognostic biomarkers and protein indicators to guide treatment decisions and clinical follow up; the current state of knowledge about the potential clinical value of gene expression profiling in ovarian cancer is discussed, focusing on three main areas: distinguishing normal ovarian tissue from ovarian tumors, identifying different subtypes of ovarian cancer and identifying cancer likely to be responsive to therapy.

In this elaborate we discuss the use of novel molecules, discovered by proteomics and genomics approaches, as potential protein biomarkers in the management of ovarian cancer, to improve the anticancer therapy for malignant ovarian tumors and to monitor the clinical follow up.

## INTRODUCTION

Despite enormous progress in cancer research, this disease represents one of the principal causes of death worldwide. For many types of cancer, the stage at the moment of diagnosis is relevant for the successive treatment; therefore, there is a need for the identification of molecules which can act as indicators of an early malignant process, when disease burden is localized. However, the possibility to use many of these molecules in the clinical practice remains, unfortunately, still elusive.

Evaluation and validation of biomarkers need rigorous procedures to ensure clinical validity.

The process follows different steps to assist researchers in developing biomarker, these phases include: preclinical exploratory studies, clinical assay and validation, retrospective longitudinal, prospective screening and cancer control [1].

In addition, genomic and proteomics technologies have increased the number of potential biomarker entering clinical development, hence the validation of these candidate biomarkers require an integrated effort among government, academic and pharmaceutical groups to coordinate the activity of many laboratories [2].

In the case of ovarian cancer, it is often reported the evidence that different histological subtypes of ovarian cancer can be distinguished by some microarray profilings, and that these subtypes might be partly reflected by a different aetiology through the deregulation and activation of different pathways.

This neoplasm heterogeneity could also account for different ovarian tumor behaviours and it could be revealed by specific biomarkers; for this evidence, it is reported by authors that gene expression profiling could be a useful prognostic tool, predicting chemosensitivity to the standard treatment combination of paclitaxel and platinum chemotherapy for advanced ovarian cancer, but more knowledge about chemosensitivity could eventually lead to a more tailored cancer therapy [1-3].

The purpose of this review is to summarize current data regarding the use of novel protein biomarkers, based on genomics and proteomics, in the diagnosis, prognosis and response to therapy in ovarian cancer. Our attention will be focus not only on serum markers but also on intracellular proteins. Indeed, the expression of these proteins can reflect cellular and molecular mechanisms that control onset and neoplastic progression. Proteins included in this group belong to different cellular pathways including cellular metabolism and signal transduction. Of particular interest, it is the role of signalling molecules that regulate interaction pathways between tumour cells and the microenvironment.

## OVARIAN CANCER: MARKERS ACTUALLY USED IN CLINIC PRACTICE

Worldwide ovarian cancer accounts for approximately 32% of gynaecological malignancies and represents 55% of female mortality associated with cancer. Widely asymptomatic at the moment of the diagnosis, more than 70% of the patients with ovarian cancer are already in an advanced disease state [3].

The serum CA125 in combination with transvaginal or transabdominal ultrasonography (US) have been helpful for clinicians in the diagnosis and monitoring of a suspicious ovarian formation, leading to their acceptance for clinical screening [4], since US is a non-invasive acceptable procedure with a large diffusion in world women screening programs and without complications.

The CA125, identified in 1981, is the only serum molecules significantly associated with ovarian cancer. Normally, serum levels are less than 35 U/ml and are increased in about 90% of patients with advanced ovarian cancer (stage III-IV) but only in 50% of women with early stage disease. This protein belongs to the protein family of mucins, a high molecular weight protein that normally coats the epithelium [5, 6]. In virtue of their localization, mucine proteins have extremely important functions in cellular physiology, like, for example, cellular adhesion. Alterations in their oligosaccharide structure have been noticed in different cancer forms [7].

CA 125 is a useful marker for the assessment of response to chemotherapy, predicting relapses and for discrimination between benign and malignant masses [8, 9].

The development of an algorithm that calculates risk of ovarian cancer, based on serial CA125 values, has shown that 20% of ovarian cancers have little or no expression of CA125 and has pointed out the importance of additional serum markers identification, as well as of new molecules capable of completing or replacing CA125 altogether, to facilitate earlier detection of ovarian cancer [10].

US in combination with CA125 has also been suggested as a potential means for early detection of ovarian cancer [11]; others studies confirmed the efficacy of this combination for the screening of general populations and high risk people [12].

For this reason, evaluation of a cystic formation with US characteristic of benignity, should lead to a normal echographic follow-up [11-13].

However, additional studies performed with echographic screening have not demonstrated adequate sensitivity and sensibility for an early diagnosis of ovarian cancer [14].

Hereby, in ovarian cancer types detecting, good results were obtained by the combination of CA125 with others serum markers; serum total inhibin levels are sensitive and specific markers of epithelial ovarian cancer in postmenopausal women; the production of inhibin by the ovaries almost stops after the menopause, but it continues from ovarian tumors [15].

After menopause, circular inhibin levels are undetectable; on the other hand, they are elevated in women with ovarian cancer [15].

Inhibin is a glycoprotein that exists as a dimeric form of two subunits (α and βA or βB), to form inhibin A and inhibin B (αβA) and B (αβB); moreover, inhibin seems to be complementary to CA125 and both markers have a total 95% of sensitivity with 95% of specificity.

Studies are directed toward establishing whether there are cancer specific inhibin forms, which may be suitable during the reproductive years, when these proteins show substantial fluctuations that would seriously compromise a preoperative assessment of any ovarian cancer [16].

## NEW PROTEIN MARKERS IN OVARIAN CANCER

Mesothelin is a cell surface protein present on normal mesothelial cells lining the body cavities and it is highly expressed in several cancers, including ovarian cancers.

Using mice lacking the *Mesothelin* gene, researchers have developed a monoclonal antibody directed against the protein and an ELISA assay for detecting serum mesothelin levels.

Elevated proteins levels have been detected in patients with mesothelioma and ovarian cancer [17]. Moreover, protein levels increase from the earlier form to more advanced cancer forms [18].

Serum mesothelin may be a useful test to monitor treatment response in mesothelin-expressing cancers, because after surgery there is a rapid decrease in mesothelin levels in patients with peritoneal mesothelioma. Future studies correlating serum mesothelin with detailed clinical information, such as tumour stage, tumour bulk, and response to treatment, will also have to be done to determine if serum mesothelin has prognostic significance and also if it can be used as a biomarker to assess response to other therapies besides surgery [17].

Important results have been obtained even in prognosis and in monitoring response to therapy. Signal Transducer and Activator of Transcription 3 (STAT3) is a latent transcription factor in several neoplastic diseases; it normally resides in the cytoplasm and can be activated thought phosphorilation by several cytokines, hormones and growth factor that play their roles using STAT3 activated pathway, regulating different biological responses, like cell development, differentiation, proliferation, motility, and survival [19].

Expression of phosphorilated STAT3 form is increased in primary ovarian cancer and its nuclear localization is associated with a poor prognosis; further studies are needed to elucidate the mechanism of activation of Stat3, its effects on downstream targets, and its role in the neoplastic transformation of epithelial ovarian cells [20].

LPAAT-β (of which we know six human isoforms) is an enzyme that catalyzes the acetylation of lysophosphatidic acid (LPA), generating phosphatidic acid (PA), acting as a second messenger in tumour cell regulation of both proliferative and survival pathways [21].

An increase of enzyme expression has been correlated to a poor prognosis and is associated with a minor survival [22]; in SK-OV-3 and IGROV1 cell lines, small interfering RNA (siRNA) was used to silence the expression of LPAAT-β reducing ovarian cancer cell lines viability [23].

LPAAT-β is an intriguing prognostic tool for the identification of high-risk epithelial ovarian cancer (EOC) and it should be a molecular target toward which to direct therapy, because of the adverse clinical outcome associated with LPAAT-β expression, even for early-stage ovarian tumours [24].

Among prognostic factors in ovarian cancers there are many members of Human Kallikrein Family (hK), of serine proteases.

This family gene is organized in a big cluster, located in 19q13.4 chromosome; different himmunohistochemistry studies have shown that hK8 could be considered a marker of positive prognosis in patients with ovarian cancer [25, 26].

In addition to hK8, extensive correlative clinical data have linked the over expression of 11 other kallikreins to ovarian cancer patient prognosis [27].

Although most reports link high kallikrein expression with poor patient prognosis (e.g., kallikreins 4, 5, 6, 7, 10, and 15), several studies also recognize some kallikreins as favourable prognostic indicators (e.g., kallikreins 9, 11, 13, and 14) [28].

These clinical findings seem to be contradictory but have been explained by authors considering that hKs and proteases in general, have a dual role during tumour progression.

Pyruvate kinase (PK) is a key enzyme in glycolysis; the isoenzyme M2-PK is shifted to a dimeric form in a variety of tumours. Since the dimeric form is over-expressed in tumour cells, it is called pyruvate kinase tumour M2 (Tu M2-PK) [29].

Tu M2-PK is present in body fluids also, most likely released from tumour cells by tumour necrosis and cell turnover. It has been previously shown for various tumours that Tu M2-PK determination in the circulation provides excellent discrimination between benign and malignant disease or may provide additional information regarding sensitivity to chemotherapy [30-32].

In a recent study of Ahmed *et al*., it has been supposed a possible link between TuM2-PK plasmatic levels and ovarian cancer malignancy showing that its concentration was significantly raised in ovarian cancer patients, particularly those with higher stage disease [33].

In another study, the same researchers have tried to determine the cut-off values for TuM2-PK sensitivity and specificity for differentiating between benign and malignant ovarian disease.

The average M2-PK concentration in cancer patients was 52 U/ml versus 27 U/ml in patients with benign conditions (p < 0.001). At a cut-off value of 22 U/ml the sensitivity of M2-PK for detecting cancer was 70% with a specificity of 65%.

However the M2-PK role in clinical practice needs further evaluation [34].

c-MET (*Mesenchymal epithelial transition factor*) is a proto-oncogene that encodes for a tyrosine kinase membrane receptor [35].

The c-Met protein is expressed mostly in in mammalian tissues and plays an important role in different cellular processes; its ligand is the HGF (*Hepatocyte Growth Factor*), known also as *Scatter Factor* (SF) and c-MET over-expression or inactivation is implicated in different tumoral disease [35].

Recently, Sawada *et al*. have analyzed c-MET role in ovarian cancer biology and its possible implication as a therapeutic target; so, it has been established the c-MET over-expression, especially in patients in higher stage disease [36].

That c-MET level could represent a potential prognostic marker for patient with higher stage EOC has already been shown by the studies of Ayhan *et al.* [37]. Sawada *et al*. provided evidence that the silencing of c-MET messenger in SKOV-3ip1 cells caused inhibition of cellular adhesion to different extra cellular matrix components and human primary mesothelial cells; in parallel a significative reduction of α-integrin and β-integrin, urokinase and matrix metalloproteinase (MMP)-2/MMP-9 activity has been observed.

In summary, this study shows that c-Met is highly expressed in a subset of ovarian cancer patients and that its inhibition can reduce adhesion, invasion, metastasis, and ultimately tumour burden. It is tempting to speculate that, after the optimal surgical debulking of a patient with ovarian cancer, consolidation therapy with a drug that targets metastasis will delay the repopulation of the peritoneal cavity by ovarian cancer cells [36].

Moreover, in 2003 Maggiora *et al*. have established a link between c-MET and RON, another member of Tyrosin-kinase receptor gene family, whose mutation is related to tumorigenesis.

It has been demonstrated that, *in vitro***, **RON and MET receptors cross-talk, synergize in intracellular signalling, and cooperate in inducing morphogenic responses; the same are significantly co-expressed in 42% (P < 0.001), hypothesizing that their action might promote ovarian cancer progression [38].

Even if many possible biomarkers are directly produced by tumoural cells, recent evidences draw our attention to tumour microenvironment, not only as an important factor in carcinogenesis, but also as a source of new markers. In fact, tumoural environment is constituted by a different cellular type system, like macrophages, fibroblasts, endothelial cells, lymphocytes, that actively attend to the tumoural progression process, through production of different molecules, measurable in biological fluids [39].

Matrix metalloproteinase (MMP) are a group of calcium-zinc dependent photolytic enzymes, capable of reducing many extra cellular matrix component; they can be produced by tumoural and stromal cells [40].

In stromal cells, high levels of MMP-2, MMP-9 e MT1-MPP, (a subgroup of no soluble enzymes, connected to cellular membrane) are linked to a poor prognosis in patients with EOC.

Although the role of MMPs as therapeutic targets remains to be further elucidated, it is possible that broad MMP inhibitors (for example, Col-3) targeting both epithelial and stromal MMPs may be useful in controlling ovarian cancer vascularization and metastasis [41].

EphA2 is a tyrosine-kinase receptor of 130KDa, belonging to Eph family; EphA2 is over-expressed in many cancers, including ovarian cancer, in which its expression is related to more aggressive cancer behaviour [42].

Interestingly,****the EphA2 gene is located on chromosome 1p36.1, which****is not only a genetic “*hot spot*” in cancer [43, 44], but also the****second most common site of complex karyotypic abnormalities****in ovarian cancer [45]. Dohn *et al. *[46] showed that EphA2 transcription is regulated by p53, a tumour suppressor protein is frequently mutated in ovarian cancer [47-49].

If further study will determine the role of p53 mutations in regulating EphA2 levels [47, 48], EphA2 may be a therapeutic target to treat ovarian carcinoma patients who have p53 mutations [49].

In addition, Hendrix *et al. *[50] and Hess *et al. *[51] have shown that EphA2 may play a role in vasculogenic mimicry.

Therefore, targeting EphA2**could have direct anti-tumour and ant vascular effects.

EphA2 role in tumorigenesis seems to concern invasion and angiogenesis process; in fact, levels of this receptor in endothelial and tumoural cells are linked to a major density of micro vessels and to an high expression of MMP-9, MMP-2, MT1-MMP [52].

Therefore, EphA2 represents an important predictor of clinical outcome and may be a potential ant vascular therapeutic target. Recently Lin *et al. *demonstrated that targeting EphA2 while concomitantly using conventional cytotoxic chemotherapy successfully decreased tumour growth *in vivo* at least in part by inducing tumour-associated endothelial cell apoptosis [53], supporting EphA2 as a therapeutic antiangiogenic target.

PDEF belong to ETS transcription factor family, characterized by a conservative domain of 85 aa that bind DNA. They are nuclear factors that participate as effectors in Ras-MAPK pathway.

The mechanisms through which they seem to play a role in tumorigenesis or in ovarian cancer progression are still unknown; these factors could alter EOC physiology, possibly in a way similar to that studied in mammalian cancer cell lines, where they significantly increase mobility and invasively.

Expression of this transcription factor has been studied, at mRNA and proteic level: data obtained show that 70% of ovarian cancer over-expressed PDEF mRNA; on the other hand, only in 33% of higher stage disease over-expressed PDEF protein [54].

Different evidences suggest a link between inflammatory process and cancer onset [55].

Many molecules produced by inflammatory tumoural or associated cells, like fibroblasts, play the role of mediator between these two processes. Cytokines are a heterogeneous protein family of low molecular weight proteins, produced by immune cells, but also by tumoural cells, that can act as stimulators or inhibitors of growth and tumour progression [56].

Interleukin 13 (IL-13) is another cytokine with anti-inflammatory activity, produced by T cells, that plays an important role in many biological activities, including cancer onset. IL-13 acts linking to its membrane receptor (IL-13R), constituted by two strands, IL13Rα1 and IL13Rα2. An alterated expression of this cytokine has been observed in tumoural tissue, in respect to normal ovarian tissue [57].

High level of α2 chain has been revealed in 44 on 53 malign ovarian cancer samples (83%) [58].

Consistent with preclinical and clinical activities of IL13 cytotoxin in phase I/II clinical trials, Kioi *et al.* [58] showed that IL-13 cytotoxin mediates antitumor activity.

One of the characteristics of ovarian cancer is their intraperitoneal (IP) spread, leading to an advanced stage of the disease invading other organs. Thus, the IP route of administration of therapeutic agents seems to be an appropriate route for disseminated ovarian cancer therapy; therefore, Kioi *et al.* investigated the efficacy of IL- 13 cytotoxin administrated IP using either a continuous infusion pump or bolus administration.

By these routes of administration, IL-13 cytotoxin mediated dramatic antitumor effects in very large established s.c. xenografted tumours [58] and therefore, IL-13 has been proposed as promising target for ovarian cancer therapy.

Higher levels of serum macrophage migration inhibitory factor (MIF) were found in ovarian cancer patients’ blood (sensibility of 77,8% and specificity of 53,3%) [59].

Moreover, studies about co culture of macrophages with cancer cell lines showed that MIF expression is induced in a JNKII and NF-kB-dependent manner and acts inducing macrophages MMP release, promoting tumoural cells invasively [60].

MIF is an inflammatory cytokine that plays an important role in regulating innate and adaptive immune responses. The potent proinflammatory effect of MIF may mediate some of the stimulatory effects of inflammation on cancer progression; so, understanding its role on the inflammatory process associated with tumour development may provide insight into the mechanism by which chronic inflammation predisposes individuals to ovarian cancer and may open new venues for targeted therapy [61].

Attempts to provide insight into mechanisms of action of MIF have suggested that the protein acts by stimulating inflammatory signals in the ascitic microenvironment such as IL-6, IL-10 and TNF-α [60].

NGAL (neutrophil gelatinase-associated lipocalin) belongs to the lipocalin protein family.

It is a protein stored in the specific granules of human neutrophils and works limiting bacterial growth at sites of infection.

Lipocalins have unusual expression in tumoural tissues, respect to normal ones.

The NGAL concentration was higher in the serum of patients with borderline grade 1 tumours respect to grade 2 and grade 3 ones and to benign tumour. More studies are needed to further evaluate the role of NGAL in the progression of ovarian carcinoma and its potential targeting in diagnosis and treatment [62].

Tumoral cells capacity to escape to immunity surveillance was proposed as one of the cancer fundamental characteristic, for the first time by Dann *et al.* [63].

One way used by cancer to escape from immunity system attach is the “immunosovvertion”, that is the immunity system suppression, through superficial or soluble specific molecules actions.

CD46 is a membrane protien that protects cells against complement-mediated toxicity.

An immunohystochemistry assay on 73 ovarian cancer biopsies showed that CD46 expression is linked to a poor prognosis [64].

RCAS1 (Receptor-binding cancer antigen expressed on SiSo cells) protein acts by inducing apoptosis in immune cells expressing the RCAS1 receptor, such as T cells and Natural Killer cells [65].

By immuno-localization studies, a high RCAS1 expression was shown in breast, gallbladder and colorectal cancers [66, 67]. Patients with uterine cancer showed higher serum levels, compared to control; moreover, RCAS1 levels are correlated to therapy response [68].

## ROLE OF THE PROTEOMICS IN THE IDENTIFICATION OF BIOMARKERS OF OVARIAN CANCER

Over the past decade, the power of high-throughput technologies has stimulated their use in many bio-medical applications. In this field, proteomics has become an important methodology in the biomarker identification. Using this method the expression of hundreds of proteins can be studied simultaneously, by taking advantage of their different biochemical properties.

This approach has been applied to ovarian cancer leading to the identification of several differentially expressed proteins related to platinum resistance. Among these, five proteins annexin A3, destrin, cofilin 1, GSTO1-1 and IDHc showed the more appreciable results, confirmed also by quantitative PCR and western blot [69].

Proteomics studies have also been applied to tumour classification by using diseased tissues and serum specimens: Bengtsson *et al.* used DIGE technology to distinguish between benign and malignant tumour groups leading to the identification of an heterogeneous groups of proteins involved in several cellular processes [70].

By coupling laser capture microdissection and 2-DE, Brown Jones *et al.* revealed that the 52 KDa FK506 binding protein, RhoG-protein dissociation inhibitor and glyoxalase I were overexpressed in invasive ovarian cancer when compared to low malignant potential ovarian tumours [71].

The immune response to tumour associated antigens represents a possible serological tool for the diagnosis of cancer.

Autoantibodies direct against tumour associated antigens presented by MHC I molecules have been identified by liquid chromatography - mass spectrometry/mass spectrometry (LC-MS/MS) using two human ovarian adenocarcinoma cell lines and serum sample from twenty patients, showing important implications in cancer detection and prognosis [72].

Recently, emerging technologies are unravelling the plasma proteome.

Surface-enhanced laser desorption and ionization with time-of-flight spectrometry (SELDI-TOF) was employed for the rapid identification of serum proteins in ovarian cancer patients.

The strength of SELDI technology is based on the selective binding of proteins on a solid-phase protein chip surface where the sample is ionized by an energy laser impulse.

The protein profile of each specimen is then analysed by software to discovery changes in the protein pattern. Petricoin *et al.* [73] analyzed the serum of unaffected controls and patients with ovarian cancers and were able to discriminate with a sensibility of 100% and a specificity of 94% all ovarian cancers in the early stage [73].

Among the possible marker identified with SELDI there was the Haptoglobin, a glycoprotein secreted by liver cells and implicated in the metabolism of hemoglobulin.

High levels of Hp-α subunit were found serum of patients with ovarian cancer.

The results of the ELISA test showed for Hp-α a 64% sensitivity and 90% specificity alone and 91% sensitivity and 95% specificity if combined with CA125.

Expression levels of 169 proteins were studied by Mor *et al.* by antibody microarray in serum of 28 healthy women, 18 women newly diagnosed with EOC and 40 women with recurrent disease.

This analysis led to the identification of four proteins: leptin, prolactin, osteopontin, and insulin-like growth factor-II.

Additional validation studies showed that the combination of the four proteins exhibited a sensitivity of 95%, a positive predictive value of 95%, a specificity of 95% and negative predictive value of 94% [74].

## CONCLUSION

Because of a lack of specific symptoms in the early phase and the limitations of existing markers for diagnostic methods, the ovarian cancer still represent, despite its low incidence, one of the leading cause of death worldwide.

Proteomics and genomics provide a central opportunity for the rapid identification and development of novel detectable biomarkers; however, the current technologies show some limitations.

Nevertheless, the study of low-abundant proteins and the need to analyze a large body of sample to extrapolate significant results, make the proteomics approach labour-intensive and the potential of a gene therapy for ovarian cancer, that involves blocking expression of specific ovarian oncogenes, seems to be far to be applied.

Currently, many genomic tests used to diagnose ovarian cancer or guide its therapy do not decrease women's risk of dying from this disease or do not improve their quality of life (QoL).

Therefore, for their rapid investigations of biological specimens mass spectrometry and protein arrays are evolving as alternative methodologies to the “*classic proteomics approach*” facilitating their use at many points of the disease management and the expanding our knowledge on biomarkers is likely to lead to more individualization of therapy and improvements in ovarian cancer prognosis.
